# Morphological alterations induced by the exposure to TiO_2_ nanoparticles in primary cortical neuron cultures and in the brain of rats

**DOI:** 10.1016/j.toxrep.2018.08.006

**Published:** 2018-08-23

**Authors:** Xavier Valentini, Pauline Deneufbourg, Paula Paci, Pascaline Rugira, Sophie Laurent, Annica Frau, Dimitri Stanicki, Laurence Ris, Denis Nonclercq

**Affiliations:** a Laboratory of Histology, University of Mons, Institute for Health Sciences and Technology, Faculty of Medicine and Pharmacy, 23, Place du Parc, B-7000 Mons, Belgium; b Laboratory of Neurosciences, University of Mons, Institute for Health Sciences and Technology, Faculty of Medicine and Pharmacy, 23, Place du Parc, B-7000 Mons, Belgium; c Laboratory of General, Organic and Biomedical Chemistry, NMR and Molecular Imaging Laboratory, University of Mons, Institute for Health Sciences and Technology, Institute of Biosciences, Faculty of Medicine and Pharmacy, 23, Place du Parc, B-7000 Mons, Belgium; d Center for Microscopy and Molecular Imaging (CMMI), B-6041 Gosselies, Belgium

**Keywords:** NPs, nanoparticles, BBB, blood-brain barrier, ROS, reactive oxygen species, MDA, malondialdehyde, NO, nitric oxide, IL-1β, interleukin-1β, TNF-α, tumor necrosis factor-α, IL-10, interleukin-10, IP, intraperitoneal, DLS, dynamic light scattering, HBSS, Hank's balanced salt solution, FBS, fetal bovine serum, BrdU, 5-Bromo-2′-deoxyuridine, SEM, standard error of the mean, MAP2, microtubule-associated protein 2, GFAP, glial fibrillary acidic protein, 4-HNE, 4-hydroxynonenal, NMDA, N-methyl-D-aspartate, NOS, nitric oxide synthase, ATP, adenosine triphosphate, CNS, central nervous system, Nanoparticles, Brain, Cell culture, Oxidative stress, Proliferation

## Abstract

Nowadays, nanoparticles (NPs) of titanium dioxide (TiO_2_) are abundantly produced. TiO_2_ NPs are present in various food products, in paints, cosmetics, sunscreens and toothpastes. However, the toxicity of TiO_2_ NPs on the central nervous system has been poorly investigated until now. The aim of this study was to evaluate the toxicity of TiO_2_ NPs on the central nervous system *in vitro* and *in vivo*. In cell cultures derived from embryonic cortical brain of rats, a significant decrease in neuroblasts was observed after 24 to 96 h of incubation with TiO_2_ NPs (5 to 20 μg/ml). This phenomenon resulted from an inhibition of neuroblast proliferation and a concomitant increase in apoptosis. In the same time, a gliosis, characterized by an increase in proliferation of astrocytes and the hypertrophy of microglial cells, occurred. The phagocytosis of TiO_2_ NPs by microgliocytes was also observed. *In vivo*, after intraperitoneal injection, the TiO_2_ NPs reached the brain through the blood brain barrier and the nanoparticles promoted various histological injuries such as cellular lysis, neuronal apoptosis, and inflammation. A reduction of astrocyte population was observed in some brain area such as plexiform zone, cerebellum and subependymal area. An oxidative stress was also detected by immunohistochemistry in neurons of hippocampus, cerebellum and in subependymal area. In conclusion, our study demonstrated clearly the toxic impact of TiO_2_ NPs on rat brain and neuronal cells and pointed about not yet referenced toxicity impacts of TiO2 such as the reduction of neuroblast proliferation both *in vitro* and *in vivo*.

## Highlights

• The NPs reach the CNS *via* the blood-brain barrier and cause inflammation and necrotic area in the white matter.
• Proliferation of glial cells and neuroblasts is affected *in vitro* and *in vivo*.
• An oxidative stress was evidenced *in vivo* in neurons of hippocampus, cerebellum and in subependymal area.

## Graphical abstract

**Figure:**
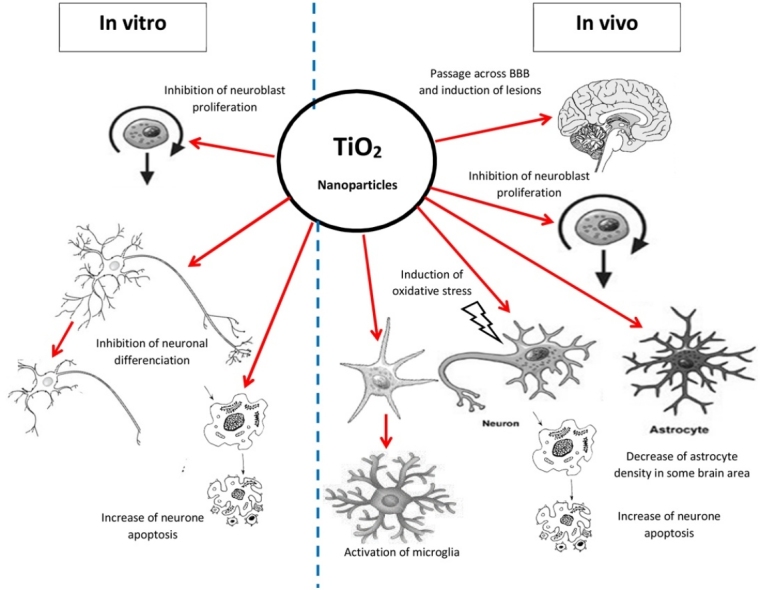

## 1 Introduction

For several years, the use of nanotechnologies, such as nanoparticles (NPs), has drastically increased in industrial and emerging countries. Inside the class of nanometric compounds, titanium dioxide (TiO_2_) NPs are one of the most produced. Since last decade, nearly 6 million tons of TiO_2_ were produced worldly [1] and the percentage of TiO_2_ under nanoform was estimated to reach 50% of the total production in the year 2023 [2]. TiO_2_ NPs are used in a large panel of uses such as cosmetic industry (sunscreens, face powder) [3], battery production, pharmaceutical industry (articulating prosthetic implants, drug coating) [4] and food industry (food coloring) [5]. The raise of utilization of NPs is due to their small size (less than 100 nm), large surface area and high reactivity [6]. Despite the wide ranges of applications, there is a lack of information about the interaction of these NPs with biological systems including the impact of TiO_2_ exposure on the nervous system at short and long time.

Many recent studies show that TiO_2_ NPs are toxic *via* different routes of exposure such as inhalation, ingestion or injection. After intraperitoneal injection in mice, the main target organs are liver, kidneys, spleen and lungs causing inflammation, fibrosis and tumors [7]. Chen et al. [8] demonstrated a toxicity of TiO_2_ NPs in rats after oral intake, which was characterized by an inflammatory process and an alteration of cardiac function. Other studies show that TiO_2_ NPs have also deleterious effects *in vitro* on different cell types including epidermal cells [9], endothelial cells [10], alveolar macrophage [11] and renal tubular cells [12] causing oxidative stress, decrease in growth and apoptosis.

Regarding the nervous system, nanoparticles have the capacity to reach various major organs including different parts of the brain *via* systemic circulation [13]. Therefore, the rise of nanotechnology and the environmental pollutants including TiO_2_ NPs may be an important risk factor of neurological disorders such as Alzheimer’s disease, Parkinson’s disease and brain tumors [14].

To reach the brain, TiO_2_ NPs have to cross the blood-brain barrier (BBB) which protects the brain from chemicals, toxins and pathogens. It is composed of tight junctions strongly connecting endothelial cells surrounded by astrocytes and pericytes. Only substances with low molecular weight are able to pass the BBB by passive diffusion, active transport or endocytosis [15]. It has been demonstrated that the intraperitoneal administration of nanoparticles (approximately 50–60 nm) derived from metals such as Ag, Al or Cu causes the disruption of neuronal cell membranes that enable their entry into the brain [16]. On the other hand, during inhalation, the NPs are directly captured by the endings bulbs of the olfactory and trigeminal nerves and can reach the brain by retrograde axonal transport [17]. Elder et al. [18] reported that manganese oxide NPs were found in rat brain after intranasal instillation by the olfactory neuronal pathway.

Reactive oxygen species (ROS) and oxidative stress have been implicated in the pathogenesis of neurodegenerative injuries. Oxidative stress is the most important accepted mechanism of nano-neurotoxicity. Inflammatory response, apoptosis, genotoxicity can be the consequences of an oxidative stress. ROS such as superoxide, hydrogen peroxide and hydroxyl radicals are able to interact with lipids, nucleic acids and proteins at the site of particle deposition. The brain is particularly vulnerable to oxidative stress because of its high energy demand, low level of antioxidants and high cellular content of lipids and proteins [19]. Several *in vitro* and *in vivo* studies demonstrated the capacity of TiO_2_ NPs to induce an oxidative stress at the site of accumulation in different parts of the brain. Shrivastava et al. [20] showed that after oral administration, ROS increased and the activities of antioxidant enzymes were disturbed inside the central nervous system. This oxidative stress was accompanied by histopathological injuries as observed after IP administration of TiO_2_ NPs [21]. Nanoparticles reached the brain and induced histopathological changes and high levels of ROS, malondialdehyde (MDA), nitric oxide (NO).

Inflammatory reaction is also a major mechanism of neurotoxicity induced by TiO_2_ NPs. TiO_2_ NPs can interact with neurons and glial cells including microglia that are immune cells residing in the brain. If microglial cells are activated by NPs, they produced pro-inflammatory cytokines that induce neuro-inflammation [22]. Liu et al. [23] (2013) have demonstrated that tracheal exposition to NPs increased significantly the expression of interleukin-1β (IL-1β), TNF-α and IL-10 in the brain. Damages in astrocytes and disruption of the BBB were also observed.

In the present study, the toxic effects of TiO_2_ NPs on the brain were investigated according to two different approaches. In the first part, *in vitro* experiments were performed on primary cortical cultures of rat embryos. The cells were exposed at different doses of NPs (0, 0.5, 10, 15, 20 μg/ml culture medium) and several times of exposure (6, 18, 24, 48, 72, 96 h) to study the effects on neurons, astrocytes and microglia. Oxidative stress and cell proliferation were also studied.

In the second part of the study, rats were exposed to NPs by IP injection (0, 0.5, 1, 4, 16 g/kg body weight). Animals were sacrificed after 4 days, 1 and 2 months. Histopathological injuries, cell proliferation and oxidative stress were investigated by immunohistochemical methods.

## 2 Material and methods

### 2.1 *In vitro* experiments

#### 2.1.1 Preparation of TiO_2_ nanoparticles suspension

TiO_2_ nanoparticles [CAS N°: 1317.70.0] were provided by Sigma-Aldrich chemical Co, (Saint-Louis, USA). According to the manufacturer specifications, the nanoparticles were composed of titanium (IV) oxide, anatase with a purity of 99.7%, based on trace of metal analysis. All suspensions were prepared in isotonic sterile solution of phosphate buffer saline (PBS). Before use, a stock solution of NPs (2 mg/ml) was sonicated in probe sonicator (UP200S, dr.Hielscher Ultrasound technology (GmbH), 50/60 Hz; 230 V) for 3 runs of 30 min as detail in a previous publication [12]. The measurements of the size distribution and the zeta potential of the nanoparticles suspended in aqueous medium were performed on a Zetasizer nano zs (Malvern Instruments, United Kingdom) using laser He-Ne (633 nm). The zeta potential was determined directly in solution containing NaCl (0.01 mM). The pH of the aqueous suspension containing the particles was adjusted by adding 0.1-0.001 mM HNO_3_ or NaOH solution.

#### 2.1.2 Cell cultures

Animals were treated according to the guidelines specified by the Animal Welfare Unit (UBEA) of the Public Service of Wallonia (agreement LA1500024) and under the control of the local UMONS-ethical commission. Gravid rats (Wistar Han) were anesthetized with an intraperitoneal injection of Nembutal (125 mg/kg), and embryos (E18-E19) were separated from uterus. Embryos were decapitated, and heads were placed immediately in iced Hank's Balanced Salt Solution (HBSS) (GIBCO® live technologies, Ghent, Belgium). Cerebral hemispheres were removed and placed in a sterile 100-mm dish containing an excess of cold dissection medium (HBSS). Under a dissecting microscope, brain hemispheres were separated and the cerebral cortices was carefully dissected removing the midbrain and meninges. Cerebral cortice samples were transferred in a sterile tube containing 2 ml of HBSS and mechanically dissociated following a procedure detailed in previous publications [24,25] After evaluation of cell density using an hemocytometer of Bürker, cell suspensions were diluted and plated at density of 7 10^4^/ 1.13 cm^2^ on sterile 12 mm diameter round glass coverslips (VWR®, Leuven, Belgium) pre-coated with polylysine in 24-well dishes (Greiner Bio-One, Vilvoorde, Belgium). Cultures were placed in an incubator at 37 °C with humid atmosphere at 5% CO_2_. Cells were fed with fresh medium (1% FBS) 2 times per weeks.

#### 2.1.3 Culture exposure to TiO_2_

A volume of 2.5–10 μl of stock suspension of TiO_2_ (2 mg/ml) was added per ml of culture medium (Neurobasal / 1% FBS) in order to obtain final concentrations of (5, 10, 15 or 20 μg/ml) in the well dishes. The cultures were exposed for different time intervals of 6, 18. 24. 72 and 96 h. Equivalent volumes of 2.5–10 μl of vehicle (PBS) were added per ml of culture medium in control cultures. The culture medium was not changed during the incubation periods.

#### 2.1.4 Evaluation of cell proliferation

Proliferating cells were evaluated in culture by immunocytochemical detection of 5-Bromo-2′-deoxyuridine (BrdU) as detailed in previous publication [25]. Briefly, culture cells were exposed to BrdU (3 μg/ml culture medium) during 2 h before cell fixation. After fixation in paraformaldehyde 4% for 15 min culture slices were rinsed in distilled water and treated for 30 min with a 3 M HCl solution at 60 °C. After rinsing in PBS, culture cells were preincubated for 20 min in a 0.01% casein solution in PBS buffer. Thereafter, cells were incubated with a mouse monoclonal anti-BrdU antibody (1:20) for 1 h at room temperature. This step was followed by an exposure of 30 min to anti-mouse/peroxidase complexes (ImmPress™ Reagent Kit; Vector, Burlingame, CA). Revelation of bound peroxidase activity was performed by incubation with a solution of 3.3′-diaminobenzidine (DAB) 0.05% and 0.02% H_2_O_2_ in PBS. Finally, culture cells were counterstained with Mayer’s hemalun and mounted in permanent medium. The number of S-phase cells was counted on 50 microscopic fields picked at random per slide at high magnification 400X representing a total scanned surface of 4.2 mm² per culture. For each time of exposure to nanoparticles and for each TiO_2_ concentration, measures were done on 4 independent cultures, 4 no-treated cultures were analyzed following a similar procedure and were used as controls. For each experimental condition, the mean was calculated on four independent cultures and data presented as histogram +/- SEM.

#### 2.1.5 Immunofluorescence microscopy

Cell monolayers present on glass coverslips were fixed with 4% paraformaldehyde in PBS. Following fixation, paraformaldehyde was changed for fresh PBS where cell cultures were stored at 4 °C until immunostaining. Before application of antibodies, cell monolayers were rinsed several times with PBS containing 0.1% Triton X-100. Before exposure to primary antibodies, cells were pre-treated for 20 min in PBS containing 0.05 M NH_4_Cl and 0.05% casein to prevent non-specific adsorption of antibodies.

Cells were exposed for 60 min to mouse-monoclonal or rabbit polyclonal primary antibodies at an optimal dilution as detailed in (Table 1). This step was followed by a 30 min-exposure to fluorescent secondary antibodies [anti-mouse IgG AlexaFluor^®^ 488 – conjugated goat antibodies or anti-rabbit IgG AlexaFluor^®^ 555 – conjugated goat antibodies] (Life Technologies Corporation, Carlsbad, USA). After final rinses in PBS, the coverslips were mounted on glass slides using commercial anti-fading medium (Vectashield^®^, Vector Laboratories). Negative controls were produced by omitting the primary antibodies. This modification resulted in a disappearance of the fluorescence signal.

**Table 1:** Antibodies used for immunohistochemistry and immunofluorescence.

Primary antibodies	Specificity	Origin	Working dilution
Anti-Microtubule Associated Protein 2 (MAP2)	Neurons and neuronal processes	Anti-MAP2 Mouse Monoclonal (Millipore, Billerica, USA)	1:100
Anti-Ionized Calcium Binding Adaptor Molecule 1 (Iba1)	Activated microglial cells	Anti-Iba1 Rabbit polyclonal (WAKO, Chemicals GmbH, Neuss, Germany)	1:100
Anti-Glial fibrillary acidic protein (GFAP)	Astrocytes	Anti-GFAP Mouse Monoclonal (BD Biosciences, Erembodegem, Belgium)	1:50
Anti-4-hydroxynonenal (4-HNE)	Compound formed by lipids peroxidation. Marker of cellular oxidative stress	Anti-4-HNE polyclonal rabbit (Abcam, Cambridge, UK)	1 :75
Anti-5-Bromo-2’-desoxyuridine (BrdU)	Marker of cells in S-Phase	Anti-BrdU (Dako, Glostrup, Denmark)	1:20

#### 2.1.6 Morphometric analysis

For each culture, the number of neurones, the length of neuronal processes and the area occupied by astrocytes were quantified by morphometric analysis at 100× magnification. The procedure utilized a software designed for morphometry and colour analysis (KS 400 Imaging system, Carl Zeiss Vision GmbH, München, Germany). For each culture condition, 5 microscopic fields were picked at random representing a total scanned surface of 1.8 mm². The number of neurones and the mean length of neuronal processes were quantified after MAP2 immunostaining. The surface occupied by astrocytes was calculated on cultures exposed to anti-GFAP immunofluorescence. For each time point, measures were done on 4 independent cultures and results were presented under box plots. Results obtained from morphometric analysis were submitted to non-parametric Mann-Whitney test (the limit of significance set at p < 0.05 by comparison to control values).

### 2.2 *In vivo* experiments

#### 2.2.1 Animals and treatment

All experiments were performed on 2-month-old male Wistar rats weighing 200–250 g originally obtained from Charles River (Belgium). Animals were treated according to the guideline specified by the Animal Welfare Unit (UBEA) of the Public Service of Wallonia (agreement LA1500021) and under the control of the local UMONS-ethical commission. Upon their arrival, the rats were transferred to an animal facility, submitted to a regular circadian cycle 12:12 h light/dark cycle. Tap water and standard rodent food were provided *ad libitum*. Experimental animals, distributed in twelve groups of 5 rats, received an intraperitoneal injection of TiO_2_ NPs prepared in normal saline (NaCl 0.9%) and administrated at four different doses (0,5; 1; 4 and 16 g/kg body weight) and were sacrificed 4 days, 1 month and 2 months after the beginning of the treatment respectively. Control groups (n = 5) received a saline injection and were sacrificed after the same time intervals. Each animal received an IP injection of BrdU (40 mg/kg BW) one hour prior to sacrifice in order to detect S-phase cells by immunohistochemistry (as detailed before). All animals were sacrificed by an overdose of Nembutal (Pentobarbital, 60 mg/ml). Just after sacrifice, brain was quickly fixed by immersion in Bouin Alcohol for 2 days. Brains were embedded in paraffin according to a standard procedure. Brain parasagittal sections were stained with Masson’s Trichrome or with Cresyl violet.

#### 2.2.2 Immunohistochemical detection of 4-hydroxynonenal (4-HNE)

Specific antigens present in the tissue were unmasked by microwave pre-treatment in 0.01 M citrate buffer (pH: 6.2) 2 × 5 min at a power of 900 W. Tissue sections were incubated overnight at 4 °C with primary antibodies [polyclonal (rabbit) anti-4-Hydroxynonenal, (Abcam, Cambridge, UK)] diluted at 1:75 in PBS. After rinsing in PBS, slices were treated with the complexe anti-rabbit/peroxidase (ImmPress™ Reagent Kit; Vector, Burlingame, CA) for 30 min at room temperature. Bound peroxidase activity was visualized by precipitation of 3,3′-diaminobenzidine 0.02% in PBS containing 0.01% H_2_O_2_. Preparation was counterstained with hemalum and luxol fast blue, dehydrated and mounted with a permanent medium. The specificity of immunolabeling was ascertained on the basis of several criteria. In each case negative controls were essayed by omitting the primary or secondary antibody or by the substitution of non-immune serum for the primary antibody. No staining was observed on these sections in these conditions.

## 3 Results

### 3.1 Physico-chemical characterization of NPs

**Fig. 1:**
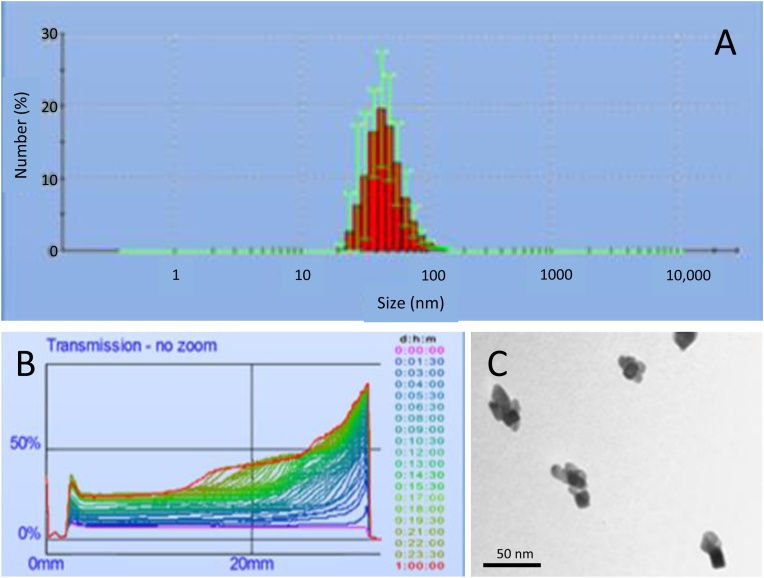
The measurements of the size distribution and the zeta potential of the nanoparticles suspended in aqueous medium were performed on a Zetasizer nano zs (Malvern Instruments, United Kingdom) using laser He-Ne (633 nm). The zeta potential was determined directly in solution containing NaCl (0.01 mM). The zeta potential of the TiO2 nanoparticles is about – 20 mV (at pH = 7). (**A**) The DLS analysis indicated that the mode and dispersion around the mode of nanoparticles was 52 ± 15 nm. (**B**) The maximal luminous transmission after 24 h for the suspension treated with sonotrod was evaluated to 32%. (**C**) Characterisation (size and shape) of nanoparticle aggregates was also performed by transmission electron microscopy (TEM). The mean size of aggregates was calculated on 10 microscopic fields picked at random. Mean nanoparticle size evaluated by this method was 34 ± 9 nm.

The average aggregate size of TiO_2_ NPs was analyzed both by electron microscopy and by dynamic light scattering (DLS), the size of NPs aggregates determined by DLS was 52 ± 15 nm (Fig.1A and B) and the mean size of the nanoparticle aggregates evaluated by electron microscopy was 34 ± 9 nm (Fig. 1C). The zeta potential of the TiO_2_ nanoparticles is about – 20 mV (at pH = 7). XPS measurements confirm that there is only titanium oxide (TiO2) and no traces of metallic titanium.

### 3.2 *In vitro* results

Primary cortical cultures of rat embryos were exposed to different doses of TiO_2_ NPs (0, 5, 10, 15, 20 μg/ml) over time periods ranging from 6 to 96 h to evaluate the effects of these NPs on neurons, astrocytes, microglia as well as their impact on oxidative stress and cell proliferation.

#### 3.2.1 Effects of TiO_2_ NPs on primary neuronal cells

**Fig. 2:**
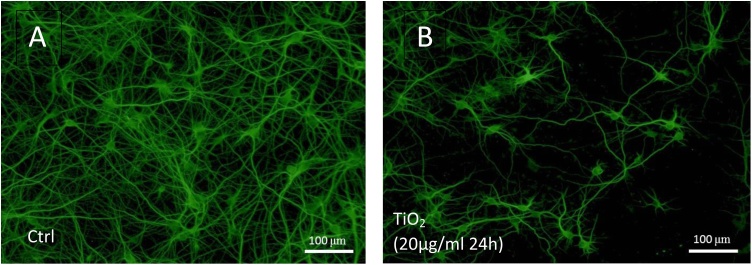
Effect of TiO_2_ exposure on primary neuronal cells demonstrated by immunofluorescence using anti-MAP2 antibodies. MAP2 labeling is localized in the dendrites and soma of neurons. The presence of TiO_2_ nanoparticles in the culture (20 μg/ml during 24 h) (**B**) induces a drastic reduction of the number of neurons and a reduction of dendritic extensions compared to controls (**A**).

To highlight neurons, MAP2, a protein that stabilizes microtubules in the dendrites was detected by immunofluorescence. An important decrease in neuronal cell density was observed in cultures exposed to TiO_2_ NPs during 24 h at 20 μg/ml (Fig. 2B) compared to control cultures (Fig. 2A). Based on the pictures taken with a fluorescence microscope, 2 parameters were quantified using a computer assisted morphometric approach: the mean number of perikaryons and the mean length of neuronal processes.

#### 3.2.2 Number of perikaryons

**Fig. 3:**
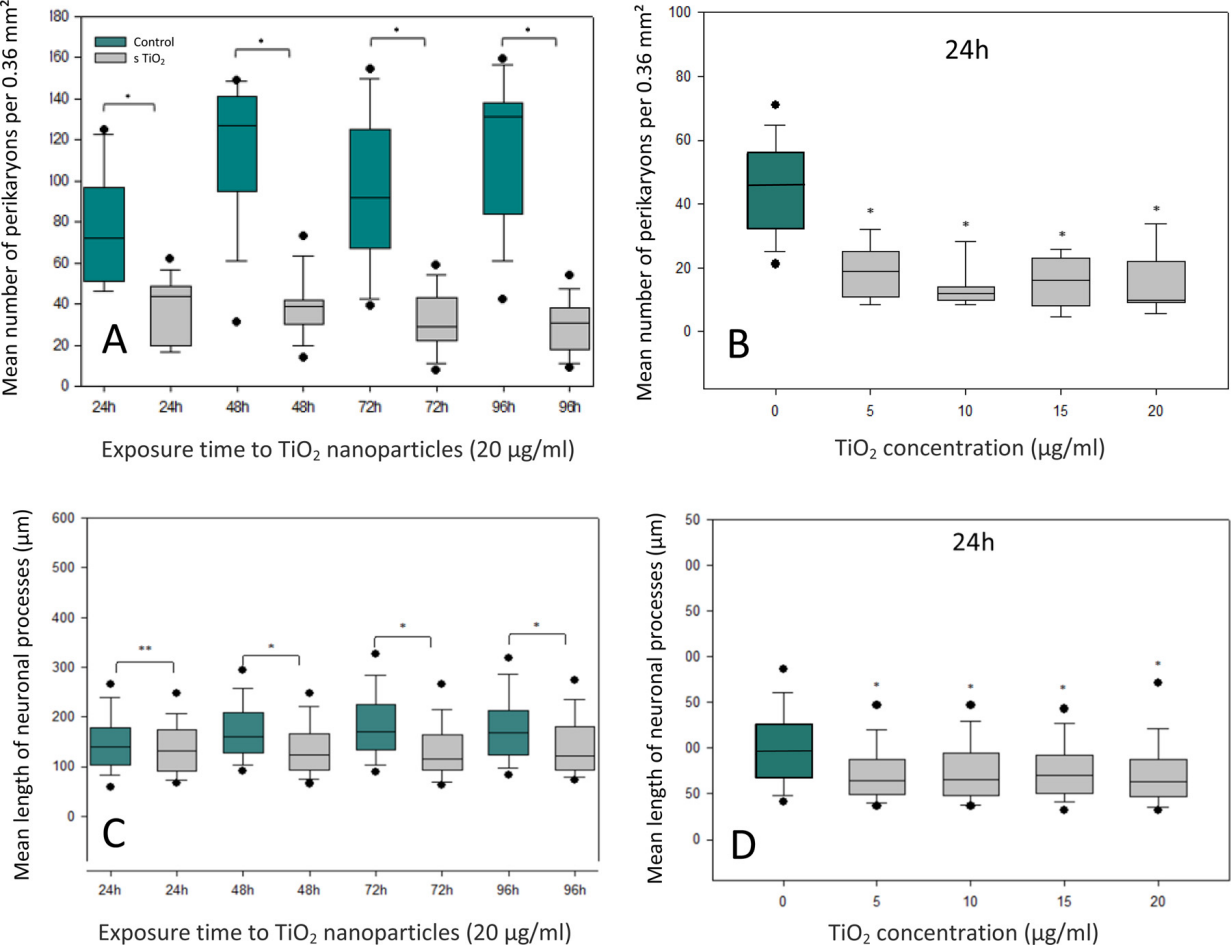
(**A**) Morphometric evaluation of the number of neuronal soma in control cultures (green box plot) and after TiO_2_ (20 μg/ml) exposure (grey boxplots) as a function of culture time. (**B**) Evolution of the number of neurons after 24 h of exposure to different doses of TiO_2_ nanoparticles. (**C**) Morphometric evaluation of the mean length of neuronal processes in control (green box plot) and after TiO_2_ (20 μg/ml) exposure (grey boxplots) as a function of culture time. (**D**) Evolution of dendritic expansions after 24 h of exposure to different doses of TiO_2_ nanoparticles. Box plot values represent the median (line), second and third quartiles (upper and lower edges of box), the upper and lower whiskers represent the 25% of inferior and superior values, excluding extrema (black points). Each measure was performed on four independent experiments (* significant values p < 0.05 *versus* control cells, Mann-Whitney test).

The effects of TiO_2_ NPs (20 μg/ml) on the number of perikaryons was assessed after 24, 48, 72 and 96 h of exposure (Fig. 3A). A significant decrease of the number of neuronal cells was observed after 24 h of exposure compared to controls. This negative effect remained constant up to 96 h. The impact of increasing doses of NPs of TiO_2_ on the density of neurons in culture was also studied (Fig. 3B). After 24 h, the number of neurons was significantly reduced compared to the control values for the different concentrations of TiO_2_. These effects do not seem to increase as a function of the TiO_2_ concentration. Indeed, the toxic effect was already observed with the lowest dose of TiO_2_ (5 μg/ml) causing a significant loss of neurons.

#### 3.2.3 Length of neuronal processes (axonal and dendritic extensions)

The length of the neuronal processes, measured using computer software, was significantly reduced after 24 h of exposure to TiO_2_ NPs (Fig. 3C) compared to controls. The gap between treated and control values increased as a function of culture times. This phenomenon was due to the fact that neurites continue to develop over time in the controls, whereas their growth was largely inhibited in TiO_2_-exposed cultures. The dose dependent study revealed that neurons exposed to the lowest dose of TiO_2_ (5 μg/ml) exhibited already a significant reduction of axonal and dendritic extension length compared to control neurons. A similar reduction was observed in all treated cultures independently of the dose of TiO_2_ present in the culture medium (Fig. 3D).

#### 3.2.4 Effects of TiO_2_ NPs on cell proliferation

**Fig. 4:**
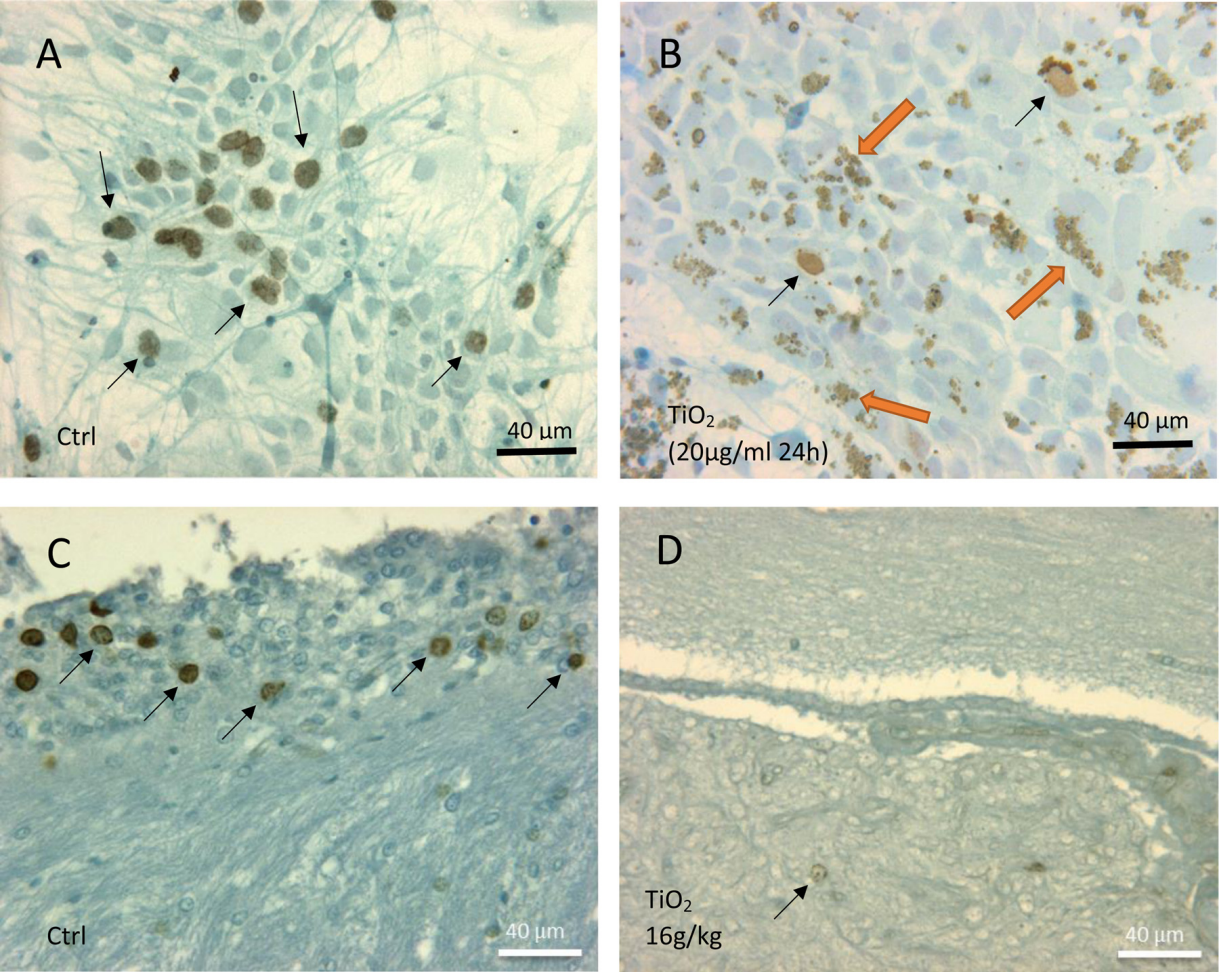
Illustration of proliferating neuroblast cells evidenced by immunohistochemistry after BrdU incorporation. (A) Control culture at 24 h presents a high level of neuroblasts in proliferation (brown nuclei pointed by arrows). (B) Drastic reduction of neuroblasts in proliferation (black arrows) in a culture exposed to TiO_2_ (20 μg/ml) during 24 h. Numerous cells present accumulation of nanoparticles aggregates inside cytoplasmic vacuoles (oranges arrows). (C) Proliferating Neuroblasts (arrows) in the neurogenic subependymal zone of a control rat. (D) Drastic reduction of cell proliferation in the subependymal neurogenesis area of a rat exposed to TiO_2_ (16 g/kg BW) and sacrificed 1 month after the treatment.

**Fig. 5:**
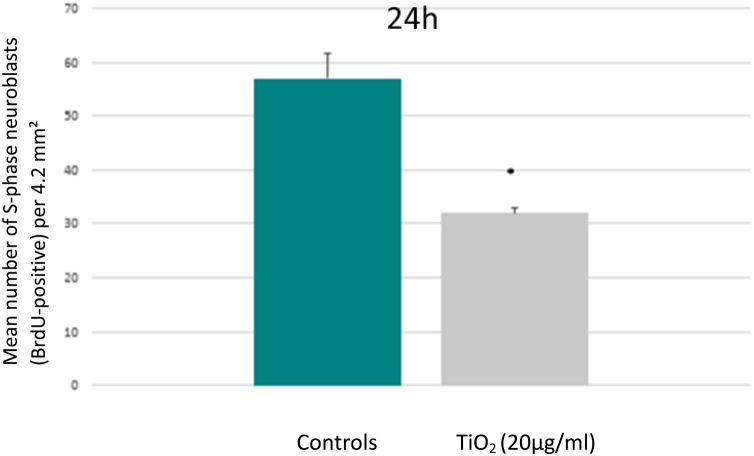
Morphometric evaluation of the number of proliferating neuroblasts in control cultures (green column) and after 24 h of exposure toTiO_2_ (20 μg/ml) (grey column). Values are presented as mean ± SD. Measure were performed in triplicate on four independent experiments (*significant values p < 0.05 *versus* controls, Student T-test).

Fig. 4A and B illustrate BrdU-positive proliferating cells in control and treated cultures respectively. By comparison to control (Fig. 4A), treated cells exhibited an accumulation of nanoparticles aggregates inside cytoplasmic inclusions distributed around nucleus (orange arrows in Fig. 4B). The proliferating cells were predominantly identified as neuroblastic type by co-immunostaining (BrdU/MAP2). Indeed, the cells used in our experimental model were derived from rat embryos and, in consequence, contained a large proportion of neuroblasts which are able to divide up to 10 days after seeding. The number of BrdU-positive neuroblasts was drastically reduced in cultures exposed to TiO_2_ (Fig. 4B) *versus* controls (Fig. 4A). These observations were confirmed by a quantitative analysis. The number of positive BrdU cells was reduced by 45% in the treated cultures (TiO_2_ 20 μg/ml; 24 h) compared to the controls (Fig. 5).

We observed the same negative impact of TiO_2_ on neuroblast proliferation *in vivo*. As illustrated in Fig. 4C, neuroblastic cells form a blastema which proliferates actively in the subependymal zone of the control rat brains. The same subependymal zone of animals exposed to a high dose of TiO_2_ showed a drastic reduction in the number of S-phase cells (Fig. 4D).

#### 3.2.5 Effects of TiO_2_ NPs on astrocytes and microglial cells

**Fig. 6:**
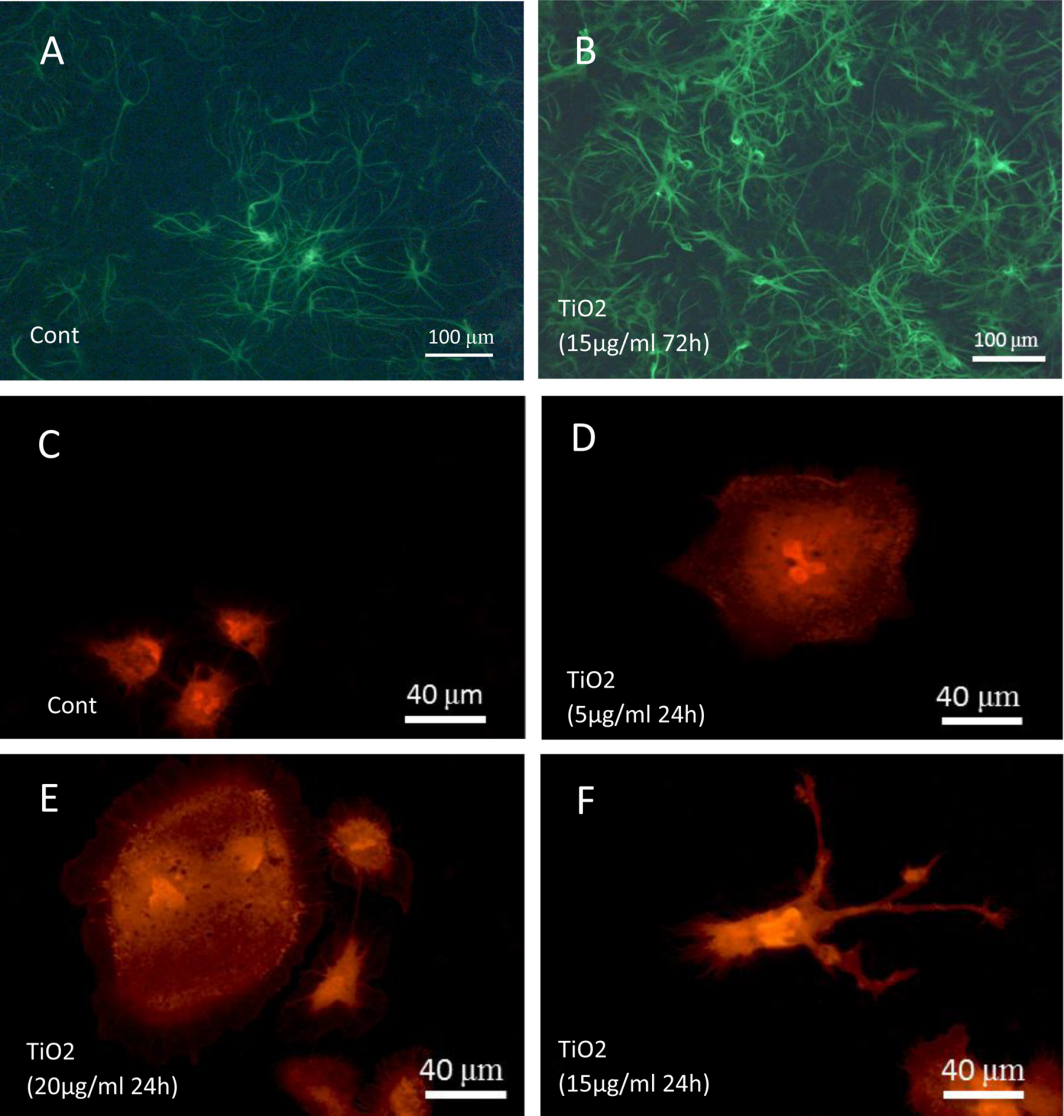
Effect of TiO_2_ exposure on astrocytes demonstrated by immunofluorescence (FITC labeling) using anti-GFAP antibodies. GFAP labeling is localized both around nucleus and in processes of astrocytes. The presence of TiO_2_ nanoparticles in the culture (15 μg/ml during 72 h) (**B**) induces an increase in astrocytes growing in the culture as compared to controls (**A**). Effect TiO_2_ exposure on microglial cells demonstrated by immunofluorescence (Texas-Red labeling) using anti-Iba1 antibodies. Microglial cells are relatively rare in control primary cultures (**C**), they present a small size and a rounded phenotype. In culture exposed to TiO_2_ nanoparticles, we observe, at low doses, an hypertrophy of microglial cells (fig **D** and **E**). At high doses, this phenomenon is accompanied by the adoption of a ramified phenotype (**F**).

**Fig. 7:**
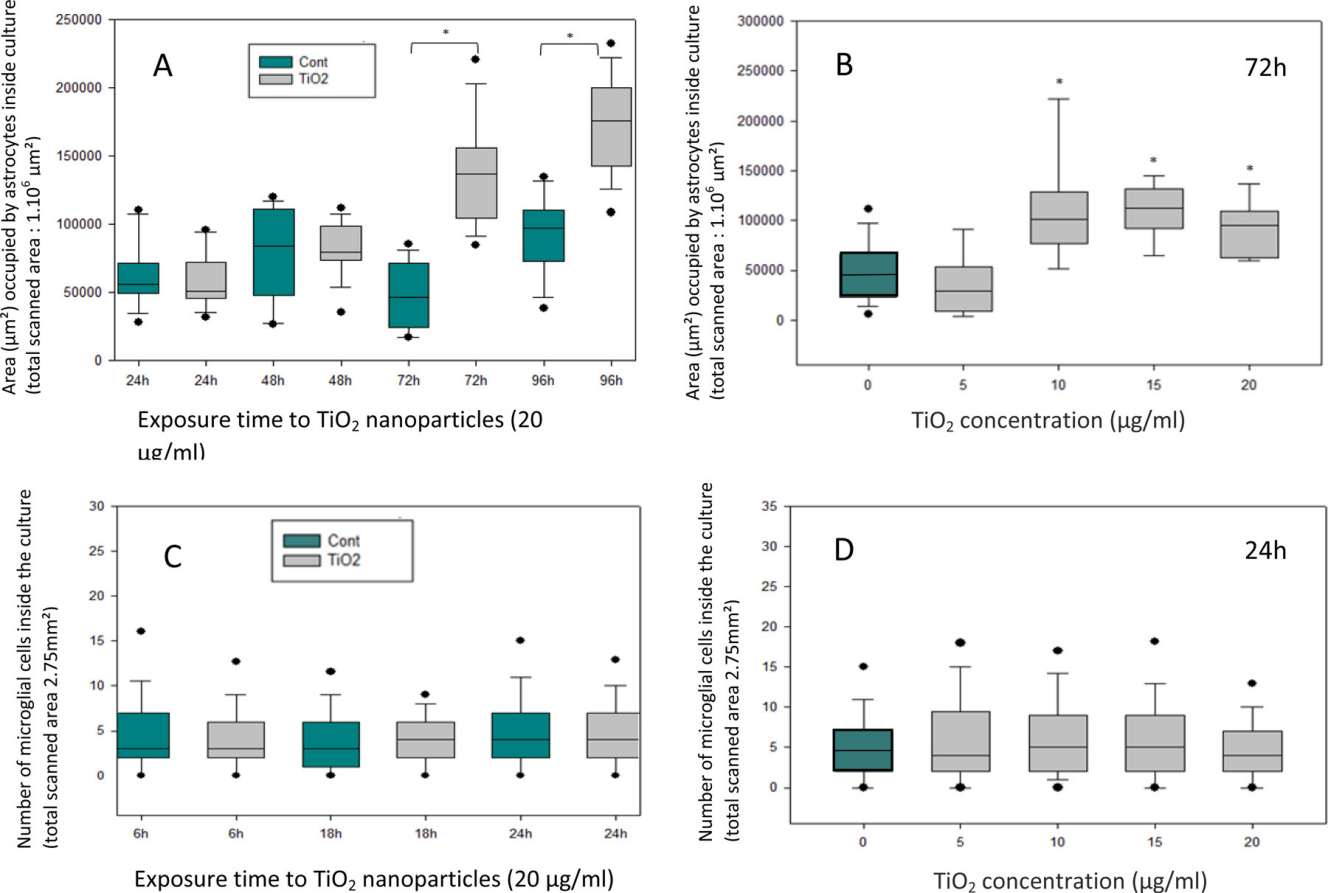
(**A**) Morphometric evaluation of the area occupied by astrocytes inside the culture in standard conditions (green box plot) or in presence of TiO_2_ nanoparticles (20 μg/ml) (grey boxplots) as a function of exposure time. (**B**) Evolution of the surface covered by astrocytes after 24 h of exposure to different doses of TiO_2_ nanoparticles. (**C**) Morphometric evaluation of number of microglial cells in control cultures (green box plot) and after TiO_2_ (20 μg/ml) exposure (grey boxplots) as a function of culture time. (**D**) Evolution of the number of microglial cells after 24 h of exposure to different doses of TiO_2_ nanoparticles. Box plot values represent the median (line), second and third quartiles (upper and lower edges of box), the upper and lower whiskers represent the 25% of inferior and superior values, excluding extrema (black points). Each measure was performed on four independent experiments (* significant values p < 0.05 *versus* control cells, Mann-Whitney test).

The protein used to target astrocytes was GFAP that is the most abundant intermediate filament of the astrocytic cytoskeleton. Immunofluorescence pictures did not show significant differences after 24 and 48 h of exposure to TiO_2_ (20 μg/ml) while a significant increase in the area occupied by astrocytes appeared after 72 and 96 h (Figs. 6B, 7 A) *versus* controls (Figs. 6A, 7 A). The dose-dependent toxic effect was also studied after 72 h. The astrocytic surface showed no difference at the lowest dose (5 μg/ml) but increased significantly for the higher doses (10, 15, 20 μg/ml) (Fig. 7B).

Microglial cells, the macrophages of the brain, have been highlighted by immunodetection of Iba1 protein. This protein is upregulated in activated microglia. No significant difference in the number of microglial cells was observed between control and treated cultures regardless of the concentration of TiO_2_ used and the exposure time (Fig. 7C and D). However, phenotypic differences were observed in microglial cells exposed to TiO_2_. Cultures exposed to NPs exhibited numerous hypertrophic microglial cells (Fig. 6D and E) that are totally absent in controls (Fig. 6C). Moreover, some microglial cells of treated cultures presented pseudopodial extensions characteristic of cell activation and phagocytosis mechanism (Fig. 6F). These phenotypic changes induced by TiO_2_ NPs expressed the transformation of quiescent microglial cells into active microglial cells.

### 3.3 *In vivo* results

The toxic effects of TiO_2_ NPs *in vivo* were realized on Wistar rats that received different doses of NPs by intraperitoneal injection. Animals were sacrificed after 4 days, 1 month and 2 months.

#### 3.3.1 Histopathological injuries

**Fig. 8:**
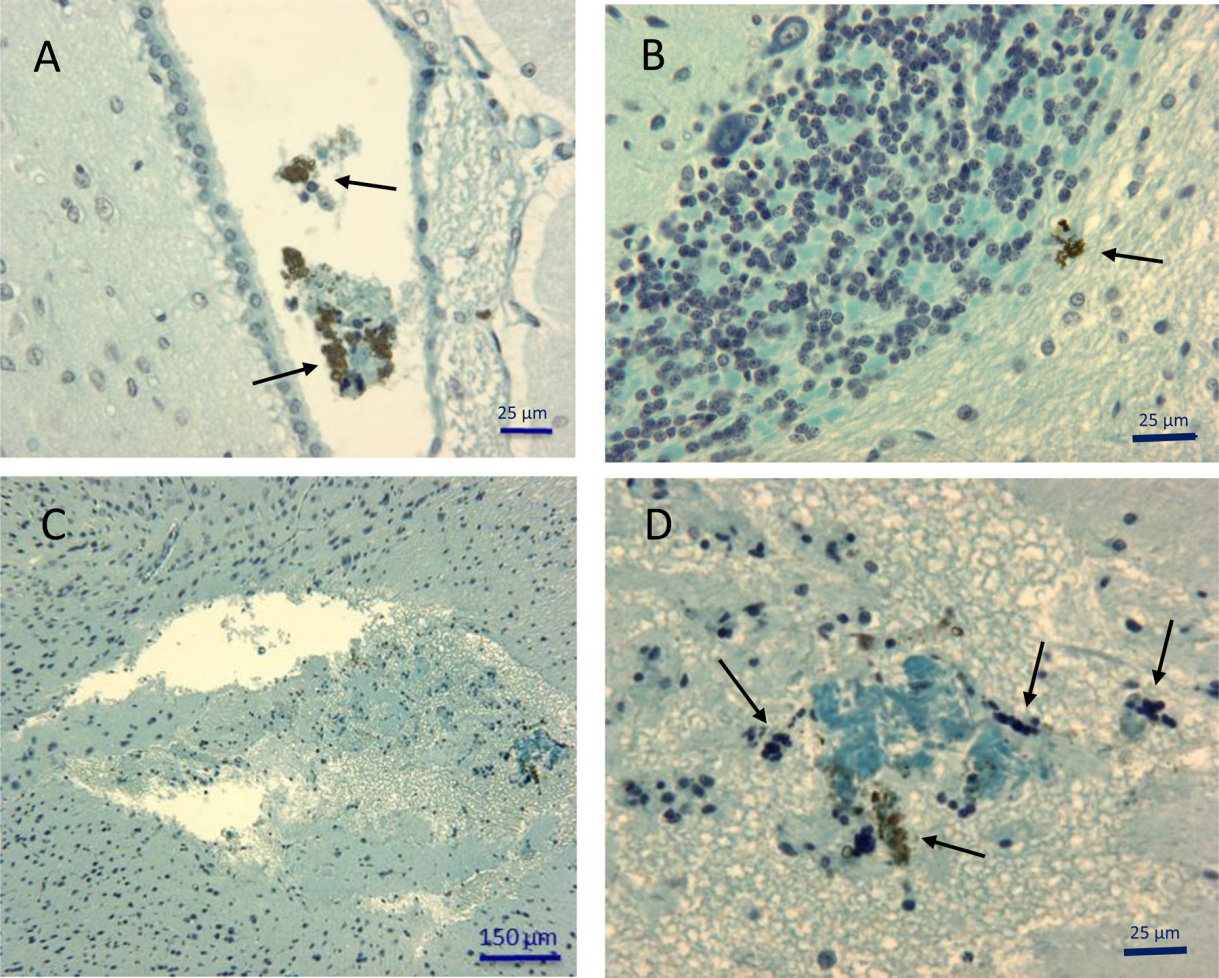
Illustration of histological alterations present in brain of rats exposed to TiO_2_ nanoparticles administrated at 16 g/kg B.W. (**A**) or 4 g/kg B.W. (**B,C,D**) and euthanized 1 month after the administration of the treatment. (**A**) Aggregates of nanoparticles (arrows) accompanied by cell fragments and inflammatory cells are present in cerebrospinal fluid inside cerebral ventricles. (**B**) Some scattered deposits of TiO_2_ are evidenced in the white matter of cerebellum. (**C**) Low magnification illustrating a large edematous and partially necrotic area in the white matter of brain. At higher magnification (**D**) the edema was accompanied by TiO_2_ aggregates (arrows) and also some fibrous material stained in blue and inflammatory cells mainly identified as lymphocytes and granulocytes.

Aggregates of NPs were observed in different regions of the brains in animals exposed to the highest doses (4, 16 g/kg BW) for 4 days, 1 and 2 months. These macroscopic aggregates of several micrometers in diameter were found in particular in the choroid plexus (Fig. 8A) and the cerebellum (Fig. 8B). In addition to the presence of aggregates, the brain of animals exposed to highest doses and sacrificed after 1 month showed areas of cell lysis localized within the white matter (Fig. 8C). These lesions were not observed in rats exposed to lower doses or shorter times. These cell necrosis areas are associated with TiO_2_ aggregates. These alterations were accompanied on one hand by the presence of numerous picnotic nuclei characteristic of apoptotic processes and on the other hand by the infiltration of polynuclear cells and lymphocytes reflecting an inflammatory response (Fig. 8D). Fibrous material accumulations were also present in these necrotic zones (Fig. 8D).

#### 3.3.2 Effects of TiO_2_ NPs on oxidative stress

Oxidative stress was assessed using an anti-4-Hydroxynonenal antibody targeting lipid peroxidation.

**Fig. 9:**
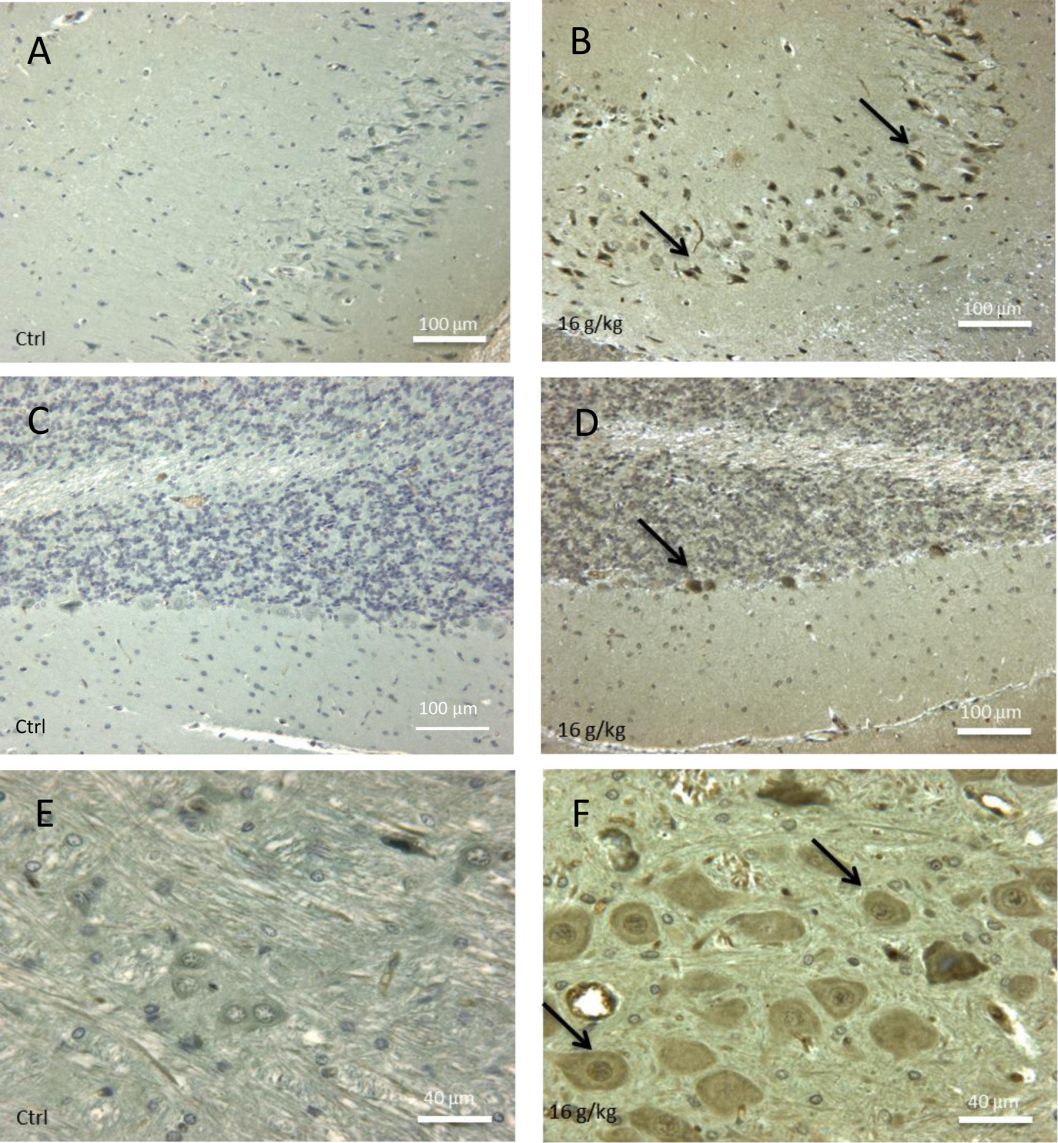
Screening of oxidative stress by immunocytochemical detection of 4-hydroxynonenal (4-HNE) in brain of controls (**A, C, E**) and in rats exposed toTiO_2_ nanoparticles (16 g/kg) and sacrificed after 1 month (**B, D, F**). In hippocampus, oxidative stress induced a production of 4-HNE (arrows) in most neurons of treated animals (**B**) as compared to the similar area of controls which is negative (**A**). In treated animals (**D**), some Purkinje cells of the cerebellum (arrow) showed an oxidative stress (arrows); by contrast, no immunoreactivity was detected in cerebellum of control rats (**C**). TiO_2_ exposed animals exhibited some large neuronal somas grouped in nuclei of white matter intensively stained by 4-HNE antibodies (**F**, arrows) whereas equivalent group of neurons in control animals are negatives (**E**).

Qualitative observations were realized in different regions of the brain. An abundant oxidative stress was evidenced in different cerebral zones of animals exposed to the high doses for 4 days and 1 month (Fig. 9B, D and F) by comparison to equivalent areas of control animals devoid of immunoreactivity (Fig. 9A, C and E). In the hippocampus, many cells were positive in the Ammon’s horn (Fig. 9B). Another region affected by oxidative stress was the cerebellum and more particularly Purkinje cells (Fig. 9D). Some of these cerebellar neurons also appeared to have a different morphological appearance from the control Purkinje cells, suggesting an apoptotic degeneration. Finally, in the sub-ependymal areas, populations of neurons characterized by large perikaryons were the site of an important oxidative stress (Fig. 9F).

#### 3.3.3 Effects of TiO_2_ NPs on astrocytes

**Fig. 10:**
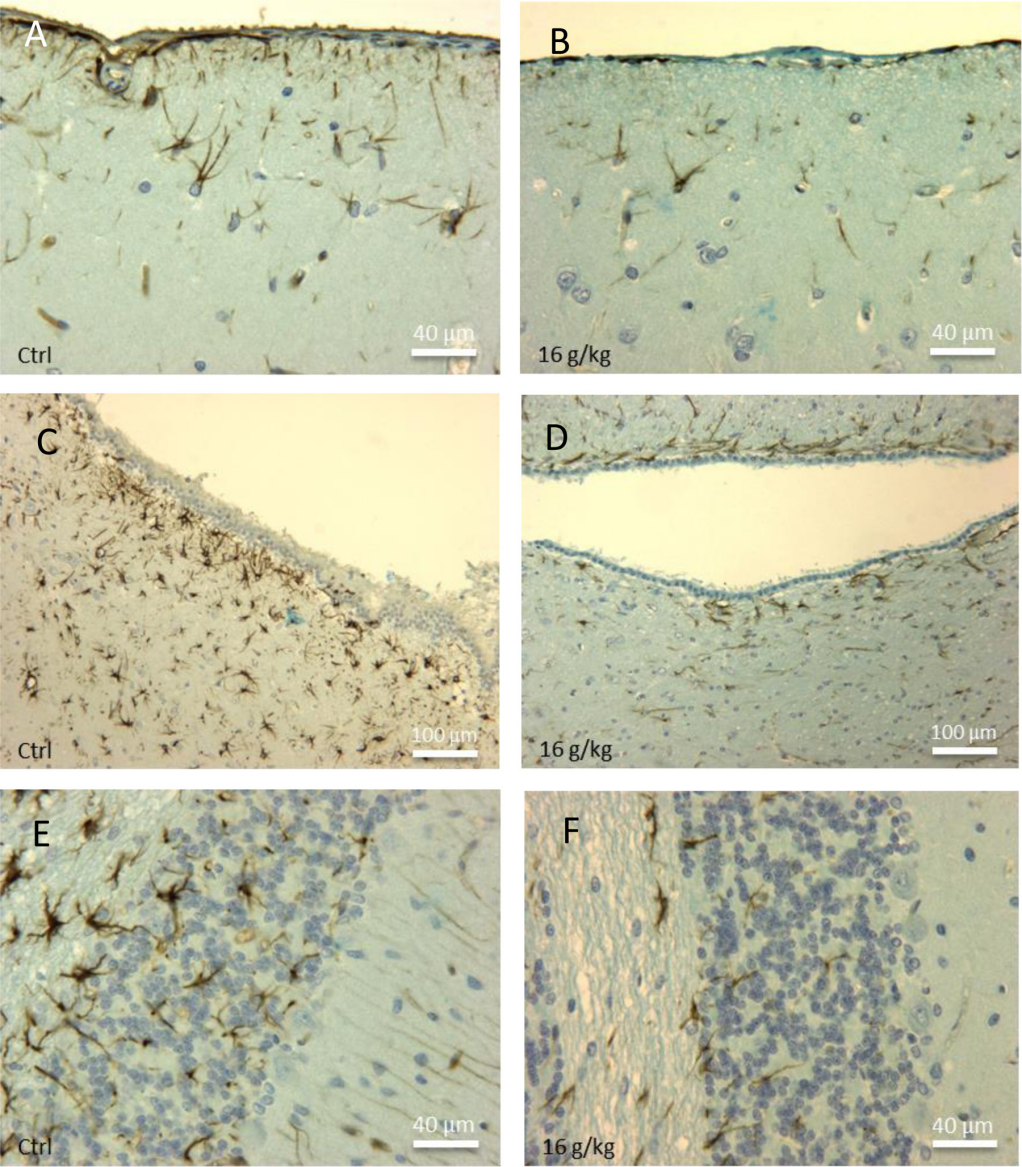
Immunohistochemical detection of astrocytes (anti-GFAP-positive cells) in different brain zones of control rats (A, C, E) and in the same area of animals exposed to TiO_2_ nanoparticles (16 g/kg BW) and sacrificed 1 month after injection (B, D, F). (A) High density of astrocytes in the plexiform zone under the pia mater of a control rat. (B) Reduction of astrocyte density in the plexiform area after exposure to nanoparticles. (C) Numerous astrocytes are present in periventricular zone of white matter in control animals; by contrast, treated rats (D) presented a drastic reduction of astrocyte population in this part of the brain. (E) Illustration of the high density of astrocyte networks in cerebellum of a control animal. (F) Drastic reduction of astrocyte number in the cerebellum of a rat exposed to TiO_2_.

Differences in astrocytic density occurred in several regions of the brain between control and treated animals with 4 and 16 g/kg BW of TiO_2_ for 1 month. There were no apparent differences in lower doses and in animals exposed to shorter times. In the plexiform zone, the astrocytic density is lower in the treated animals (Fig.10B) *versus* controls (Fig. 10A). Many astrocytes were also present in the sub-ependymal space bordering the cerebral ventricles of the control rats (Fig. 10C). A significant decrease in the number of astrocytes in this area was observed in the treated animals (Fig. 10D). Finally, a high density of astrocytes was immuno-detected in the white matter of the cerebellum of the control rats (Fig. 10E). TiO_2_ induced also a strong reduction in the astrocytic population in that zone of the cerebellum (Fig. 10F). All of these observations reflected a general decline in the number of astrocytes in animals exposed to NPs.

## 4 Discussion

In recent years, TiO_2_ NPs have been widely used in a large panel of industrial products such as candies, toothpastes, pharmaceutical excipients, paper, paints and sunscreens. Despite the increase in the use of NPs, there is a lack of information on the impact of NPs on the environment and human health. Several *in vitro* and *in vivo* studies have demonstrated toxic effects associated with exposure to TiO_2_ NPs. Liu et al. [26] observed inflammatory injuries in lungs following exposure to TiO_2_ NPs. Dysfunctions and histopathological injuries were also observed in kidneys, liver, spleen when animals were exposed to NPs [27]. However, few studies have investigated the toxic effects of these nanoparticles on the central nervous system.

The exposure of humans to TiO_2_ *via* different consumer products is estimated at 5 mg per person per day. This represents a quotidian dose of 0.07 mg/kg body weight [28]. The toxic effect could result from the cumulative effect of this compound that are not efficiently eliminated once incorporated into cells and tissues. Indeed, the presence of TiO_2_ aggregates in the brains of rats one and two months after the injection demonstrates the absence of elimination of these particles after their incorporation in the brain. The doses used in the present study were in the range of those most often mentioned in the literature for *in vitro* [[29], [30], [31], [32], [33]] and *in vivo* experiments [27,[35], [36], [37]].

Our study attested that NPs have the ability to cross the blood-brain barrier. Macroscopic aggregates of TiO_2_ particles were observed in the central nervous system of rats exposed to high doses of TiO_2_ NPs administered by intraperitoneal injection. These aggregates have been found in different brain regions such as cerebellum, choroid plexus, hippocampus, and white matter. In the white matter, these TiO_2_ accumulations were associated with areas of tissue necrosis and inflammation. Such inflammatory phenomena have been described in the hippocampus of mice exposed to TiO_2_ [21] with overexpression of different cytokines such as TNF-α and IL-1β. These inflammatory mediators could be released by activated microglial cells [38].

Our *in vitro* approach has revealed a deleterious effect of NPs on neuronal cells. Nanoparticles were internalized in neuroblasts present in culture and induced a drastic decrease in the number of perikaryons already after 6 h of exposure. These results were in accordance with similar studies mentioned in the literature. Indeed, Hong et al. [39] have demonstrated that TiO_2_ NPs can be internalized by hippocampal neurons of rats *in vitro* and were distributed in the cell nucleus inducing oxidative stress and apoptosis [34,40].

Neurite outgrowth is an important process in brain development and is associated with the synaptic structure, characteristics of information transmission efficiency and neuronal synaptic plasticity. We have clearly evidenced a significant reduction in the growth of axonal and dendritic extensions in primary neuron cultures. This phenomenon can lead to a reduction in memory and learning abilities [39].

Oxidative stress characterized by increased ROS production is recognized as the main mechanism of toxicity induced by TiO_2_ NPs [41]. An oxidative stress was detected by immunohistochemistry *in vivo* in rats who received the highest doses of NPs. The oxidative stress, evidenced by an anti-4-Hydroxynonenal antibody targeting lipid peroxidation, was present in neuronal populations of different cerebral zones including cerebellum and hippocampus. The hippocampus was particularly affected both at the level of the Ammon’s horn and of the dentate gyrus.

By studying the rate of cell proliferation in cultures derived from cerebral cortex of rat embryos, we have evidenced a significant decrease in the number of dividing neuroblasts in cultures exposed to TiO_2_. Neuroblasts are stem cells able to differentiate into neurons during brain development. These cells have the ability to divide in primary embryonic brain culture several days after seeding [42]. *In vivo*, BrdU positive cells were detected in the subventricular zone of control animals known to be one of the few areas to still harbor stem cells in adults. The proliferation of neural stem cells in this zone is inhibited in the presence of TiO_2_. Two hypotheses can be raised regarding the decrease of S-phase cells in treated animals. The first is that TiO_2_ NPs could interfere with the capture of BrdU from blood, which could explain the decrease in the number of BrdU positive cells. However, obtaining similar results *in vitro* favors a second hypothesis which consists in an inhibition of the division capacities of the cells related to exposure to TiO_2_. This inhibition could result from a perturbation of the enzymes involved in the control of the cell cycle and DNA replication process [31].

The toxicity in the neuroblastic cells underlines the risk linked to the TiO_2_ NPs on the proliferation and the differentiation of these cells during the cerebral development. Takeda et al. [43] exposed pregnant mice with TiO_2_ and found NPs in the brain and testicles of newborn mice. These data indicate their ability to cross the placental barrier. The fetuses do not yet possess all the defenses present in adults, such as, for example, the blood-brain barrier which is still immature at this stage. The impact of NPs on the ability of division of neuroblasts could have very detrimental consequences on cerebral development.

In the present study, two types of glial cells were also studied: astrocytes and microglial cells. Astrocytes are the most abundant glial cells in the CNS. The increase in the area of the astrocytic network in the cultures exposed to nanoparticles at short term can be explained by glial cell activation leading to a rapid growth factor liberation in response to a general stress of the culture in the presence of TiO_2_. The massive death of neuroblastic cells in TiO_2_-exposed cultures may also explain a larger extension of the astrocytes which are more resistant to the toxic action of TiO_2_ and which could take the space left in the culture by the rapid disappearance of neuroblasts.

By contrast, our *in vivo* study points to a significant decrease in astrocyte density in several cerebral areas such as plexiform zone, cerebellum and sub-ependymal space. In the long term (1 month or 2 months of exposure), NPs could induce toxicity to these glial cells leading to a massive apoptotic process as suggested by Liu et al. [23]. The decrease in the number of astrocytes observed after 1 month could also result from the decrease in the rate of proliferation that affects the glial cells of animals exposed to TiO_2_ as suggested by Márquez-Ramírez [32].

Microglia cells are the resident macrophage-like cells in the CNS that play a pivotal role in the brain’s innate immunity [44] (Henn et al. 2009). If pathogens or exogenous elements such as metallic nanoparticles are introduced in the brain, microglia responds to this invading to prevent neuronal damages [45]. Morphological changes of these cells occurred in cultures exposed to TiO_2_ NPs. A larger size and formation of membrane protrusions typical of phagocytosis were detected. These phenotypes correspond to microglial cell activation caused by the presence of TiO_2_ NPs. When these cells are activated, they can release mediators that act on other cell types such as astrocytes [29]. The release of these cytokines could explain the activation of astrocytes and their increased proliferation in TiO_2_ exposed cultures.

In conclusion, TiO_2_ NPs clearly demonstrate a toxic effect on CNS. The NPs have the ability to cross the BBB. Immunohistochemical analyzes show oxidative stress detected in several types of neuronal cells. This toxicity is marked *in vitro* by a significant reduction in the number of neurons and the size of their neurites as well as an activation of the microglial cells. Inhibition of neuroblast proliferation has also been demonstrated in both *in vitro* and *in vivo* studies. The effects of TiO_2_ NPs on CNS are not limited to neurons alone but also affect astrocytes and microglia.

## Conflict of interest

I attest that our article presents no potential conflict of interest (Prof. Denis NONCLERCQ).
